# Mastering Snow Analysis: Enhancing Sampling Techniques and Introducing ACF Extraction Method with Applications in Svalbard

**DOI:** 10.3390/molecules29215111

**Published:** 2024-10-29

**Authors:** Marina Cerasa, Catia Balducci, Benedetta Giannelli Moneta, Ettore Guerriero, Maria Luisa Feo, Alessandro Bacaloni, Silvia Mosca

**Affiliations:** 1 Institute for Atmospheric Pollution Research, Italian National Research Council (CNR-IIA), c/o Area della Ricerca di Roma1, Strada Provinciale 35d n. 9, Montelibretti, 00010 Rome, Italy; catia.balducci@cnr.it (C.B.); benedettagiannellimoneta@cnr.it (B.G.M.); ettore.guerriero@cnr.it (E.G.); marialuisa.feo@cnr.it (M.L.F.); silvia.mosca@cnr.it (S.M.); 2 Department of Chemistry, “Sapienza” University of Rome, Piazzale Aldo Moro 5, 00185 Rome, Italy; alessandro.bacaloni@uniroma1.it

**Keywords:** snow analysis, activated carbon fibers, Svalbard contamination, POPs, sampling techniques, extraction method

## Abstract

Semi-volatile organic contaminants (SVOCs) are known for their tendency to evaporate from source regions and undergo atmospheric transport to distant areas. Cold condensation intensifies dry deposition, particle deposition, and scavenging by snow and rain, allowing SVOCs to move from the atmosphere into terrestrial and aquatic ecosystems in alpine and polar regions. However, no standardized methods exist for the sampling, laboratory processing, and instrumental analysis of persistent organic pollutants (POPs) in snow. The lack of reference methods makes these steps highly variable and prone to errors. This study critically reviews the existing literature to highlight the key challenges in the sampling phase, aiming to develop a reliable, consistent, and easily reproducible technique. The goal is to simplify this crucial step of the analysis, allowing data to be shared more effectively through standardized methods, minimizing errors. Additionally, an innovative method for laboratory processing is introduced, which uses activated carbon fibers (ACFs) as adsorbents, streamlining the analysis process. The extraction method is applied to analyze polychlorobiphenyls (PCBs) and chlorinated pesticides (α-HCH, γ-HCH, p,p′-DDE, o,p′-DDT, HCB, and PeCB). The entire procedure, from sampling to instrumental analysis, is subsequently tested on snow samples collected on the Svalbard Islands. To validate the efficiency of the new extraction system, quality control measures based on the EPA methods 1668B and 1699 for aqueous methods are employed. This study presents a new, reliable method that covers both sampling and lab analysis, tailored for detecting POPs in snow.

## 1. Introduction

Persistent organic pollutants (POPs) are removed from the atmosphere by wet and dry deposition. Wet deposition occurs through snow and rain, whereas dry deposition involves particulate matter and the gaseous phase. The scavenging of contaminants from the atmosphere results from a combination of both processes, which vary according to the physical and chemical properties of contaminants [1]. Semi-volatile organic compounds (SVOCs), which represent the majority of POPs, can be found both in the vapor phase and adsorbed on particulate matter. The greater affinity of a chemical species for a deposition pathway depends on the properties of the species, its concentration, the environmental conditions, and the amount of the depositions (wet and/or dry). To understand this process, it is important to know (1) the distribution of chemical species between the two phases, and (2) the prevailing deposition process as a function of the distribution. Wania and Lei (2004) showed that snow is the most effective in scavenging chemical products among all deposition types [2]. During snowfall, gaseous compounds are removed from the atmosphere, and aerosol particles can be incorporated into snowflakes until final deposition. Furthermore, all the phases should be considered a single system because particulate matter constitutes the nucleation center for snowflakes (and raindrops) in the atmosphere. For this reason, the composition of snow cover or ice, including the compounds adsorbed on the particulate matter and by the snowflakes themselves, indirectly reflects the actual state of the atmosphere in terms of pollution levels [3,4].

Organic chemicals associated to human activity (i.e., pesticides, polychlorinated biphenyls, and dioxins) can be found in remote areas due to long-range atmospheric transport (LRAT) from the continents [5,6,7] and a series of evaporation, condensation, and desorption from the particulate matter (the so-called “grasshopper effect”). After LRAT, snow acts as an effective scavenger and a deposition medium for atmospheric POPs (both gas and particle phases) [2,8,9]. Given the scavenging capability of snow, cold areas act as sinks for organic pollutants in a process known as “cold-trapping” [6,10,11,12]. During winters, the structure of snowflakes is altered by the pressure from the upper layers of snow after deposition, and together with temperature changes, especially those linked to spring, this process can concentrate contaminants that are not released until the snow melts. Therefore, in the summer season and with snowmelt in general, POPs are released into the environment (e.g., soil, water bodies) [13,14].

Certainly, this process has implications that emerge experimentally; Franz’s study [15] compared the concentration of pollutants during parallel sampling in the atmosphere and melted snow. The study shows that after collecting the snow sample, new distribution equilibria of pollutants (primarily related to hydrophobicity) are established among meltwater, residual snow, and particulate matter, similar to what happens in spring at high latitudes and altitudes. Franz concluded that in the samples, the process of pollution scavenging from the atmosphere carried out by the particulate matter and snow cannot be distinguished unless the kinetics of the adsorption and desorption of pollutants from/to the particulate matter in cold water are slow enough during the melting phase [9].

From what we have discussed so far, a snow sample collected for SVOC analysis consists of three components: the liquid fraction of melted snow (meltwater), the particulate matter, and the vapor fraction, which also includes the compounds present in volatile form (corresponding to the headspace of the sample vessel) [16,17]. These should be combined to determine the total concentration of a given compound.

Another aspect to be considered is the chemical and physical behavior of each class of POPs. The n-Octanol/Water Partition Coefficient (KOW) describes the solubility in water and the distribution of each chemical [18], i.e., it indicates how much an organic compound tends to accumulate in the lipid fraction rather than in the aqueous phase. According to the literature, compounds with a KOW greater than 3.9 should be considered dangerous for the environment since their intake/accumulation is higher than their elimination [19]. The most “water-soluble” compounds with low KOW values, such as HCHs, can be eluted directly with the first melted snow. Less soluble or hydrophobic compounds, e.g., PCBs (polychlorinated biphenyls) and DDT [18,20], enrich the remaining fraction of the snowpack, which includes the particulate matter [9]. Analytical challenges arise in detecting these substances because, unlike other matrices like air, water, and rain, snow does not have a standardized and well-established procedure for sampling and extraction of POPs, preventing the intercomparison of the data.

Sampling is a step that is often underestimated in most studies. However, this phase can introduce numerous errors, contaminations, and sample degradations if not performed correctly. As a result, the sample may become unrepresentative, and no matter how impeccably the subsequent steps are carried out, improper sampling can compromise all efforts and invalidate the results of the entire analysis.

Literature reviews show that snow is treated in different ways depending on the class of pollutants being investigated. Parameters such as temperature and altitude are constantly recorded and sometimes integrated with the stratigraphic data of the snowpack, such as snow density and crystal morphology. Moreover, GPS coordinates and the sampling area (length, width, and depth) are usually recorded [21]. A common protocol for snow sampling is the collection of surface snow: samples are collected manually with a pre-cleaned shovel or through other techniques that are often not specified [21,22,23]. Samples should ideally be collected downwind, and operators should wear powder-free latex gloves to avoid direct contamination of the snowpack, even if this is not always possible [24,25]. There is no standardized procedure for the depth of snow collection: in some studies, the first layer (3 cm) is excluded, while in others it is collected [21,26]. The collection volume varies greatly, depending on the type of site and the expected concentration of contaminants. If the site is close to the emission sources of the pollutant class taken into consideration (e.g., rural or urban area), it is advisable to collect volumes of snow that yield 1–2 L of melted snow [21,25]. However, the investigations in remote areas require larger volumes of melted snow (between 10 and 40 L), strictly connected to the detection limits of the analytical technique adopted [6], especially if it includes the analysis of trace compounds. Defining standardized container volumes for melted snow is also challenging since the volume depends on the snow texture. There is inconsistency in the selection of container materials for the same class of pollutants, and cleaning procedures are not always included. Options may range from aluminum or steel drums to pre-washed amber bottles, Teflon bottles or bags, and polyethylene cans or bottles [21,27,28]. Given the complexity of the snow system already highlighted above, the “material” of the collection container should not be neglected, since the distribution between one phase and another of the species under investigation can vary, leading to underestimation or overestimation of the results [29]. After collection, the samples are transported to a laboratory covered with a light aluminum foil to prevent photochemical interactions with short-wave radiation [24] and melted for the analysis. There are no universally accepted guidelines for snow melting, and the methods employed can differ among research groups. For instance, Talovskaya et al. conducted their melting process at room temperature for 24 h [30], others at 60 °C for 20 to 40 min [31]. In some cases, the melting phase was carried out in a dark room [26]. This step is prone to errors due to the volatility of the compounds during the snow melting procedure, especially for the lighter compounds with a higher Henry’s law constant, as evidenced in the studies of Herbert [27]. Since there are no defined procedures for extracting POPs from snow, it can be assumed that once the snow has melted, the sample can be treated as rainwater.

The second step following snow sampling, and the focus of this work, is the extraction of the investigated congeners from the matrix.

Considering meltwater as the matrix instead of snow, it can be stated that the extraction of compounds from water can be performed in two different ways, as follows: (1) direct extraction of compounds from water using a selected solvent (liquid/liquid extraction, LLE) or (2) extraction of an adsorbent medium used to trap compounds by filtering the water (solid phase extraction, SPE). The LLE method, though the least used, is the most straightforward of these techniques and is recommended in some EPA reference methods [32]. SPE, on the other hand, is faster than LLE, although it can present issues like cartridge clogging with samples containing large amounts of organic material and particulate matter, as in wet depositions. In SPE, there are several stationary phases, among which C18 and Florisil are the most common in the analysis of PAHs [27,33]. SPE cartridges with the HLB adsorbent, consisting of a specific ratio of two monomers (the hydrophilic N-vinylpyrrolidone and the lipophilic divinylbenzene) [34], are also widely used.

As mentioned, there is no standardized and well-established procedure for sampling and extraction of POPs from snow, preventing the intercomparison of the data. The fact that nearly every research group uses a different procedure significantly limits the potential for developing a monitoring network that facilitates data sharing. This is because each procedure introduces distinct types of errors, making it difficult to compare data from different studies.

This work presents two important results for the analysis of POPs in the snow matrix.

First of all, a sampling procedure that can be performed and is valid for all POPs in snow is given. Since there are no univocal procedures for snow sampling, the methodologies found in the literature have been critically examined to extrapolate all the precautions and to unify everything while considering interferences and contaminations.

Secondly, an extraction method for the total quantification of the fraction present on the particulate matter (adsorbed) and dispersed in the liquid matrix (meltwater) is presented. In detail, activated carbon fibers (ACFs) are used as adsorbents, focusing the analysis on PCBs and chlorinated pesticides (α-HCH, γ-HCH, p,p′-DDE, o,p-DDT, and HCB). ACF is a very strong adsorbent, and its effectiveness as a passive adsorbent in water and air sampling for PCDD/Fs and PCBs has already been demonstrated in other studies [35,36]. For this purpose, the solid-phase extraction with ACF (SPE-ACF) was validated in accordance with the EPA Method 1699 for pesticides and the EPA Method 1668B for PCBs in water [32,37]. Given the absence of a specific method for snow sampling and extraction, each step was carefully considered, critically justified, and motivated. This is especially true for the extraction process, since EPA methods require justification for any variations.

## 2. Results and Discussion

**Figure 1:**
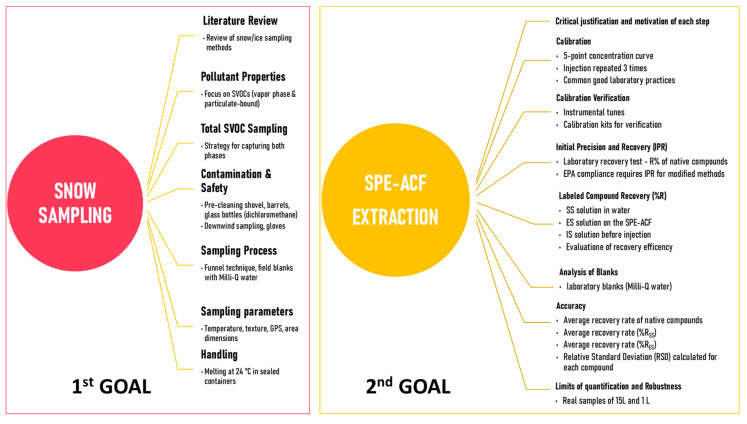
Objectives of the work: snow sampling on the left and SPE-ACF extraction on right.

Figure 1 illustrates the two key processes central to this study. On the left, the snow sampling workflow is outlined, where each line highlights critical considerations to ensure the integrity of the snow samples for subsequent analysis. The procedures for validating the SPE-ACF extraction method are shown in the figure on the right. Efficiency tests, parameter optimization, and solvent selection are all part of this process, which is carried out in compliance with the stringent guidelines of EPA methods 1699 and 1668B. Every stage is intended to verify the precision and repeatability of the procedure in removing contaminants from the snow samples.

### 2.1. Snow Sampling—Collection and Handling Method

Snow/ice samples (deposited and subsequently frozen snow) were collected using the shovel technique. To this end, an aluminum shovel, four 50 L stainless steel barrels, and four 1 L glass bottles were pre-cleaned with dichloromethane and then used to collect the snow samples via a stainless-steel funnel. Two 1 L glass bottles were also cleaned for the field blanks. Samples were collected downwind, and operators wore powder-free latex gloves to avoid direct contamination of the sample. The snowpack surface and depth temperatures, texture, GPS locations, and dimensions of the sampling area were all measured and documented (Table S2). Figure 1 (left) presents a schematic summary of the process that led to defining the best procedure for sampling. The samples were left to melt at room temperature (24 ± 1 °C) in a gas-tight sample container for two days.

### 2.2. SPE-ACF Extraction—Custom Design

In accordance with the EPA 1699 and 1668B methods, each modification to the reference extraction system is justified in the following paragraphs. Below, the extraction method developed is described and justified step by step.

**Figure 2:**
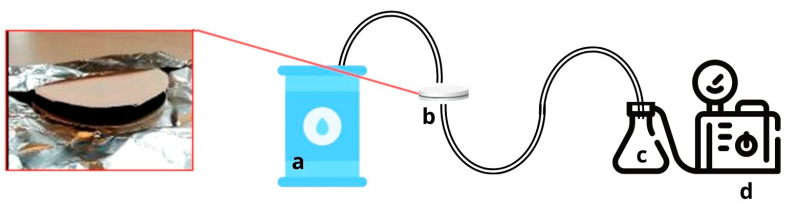
Custom-designed extraction system for ACF-based SPE extraction: (**a**) Sample container (50 L or 1 L container); (**b**) Holder 102 mm for the SPE-ACF (QFF/ACF/QFF) shown on the left; (**c**) Vacuum flask; (**d**) Pump.

The extraction was carried out on an aqueous matrix (the snow was melted as described in the previous section). The sample was then filtered through a custom-designed sandwich solid-phase extraction (SPE) system that includes an ACF filter [35,36,38]. The SPE-ACF arrangement required the adsorbent to be placed between two quartz fiber filters (QFF/ACF/QFF) (Figure 2). This configuration allowed the first QFF to be replaced if clogged with particulates.

Before starting the water filtration, the SS solution was mixed with acetone and spiked into the melted snow, while the SPE-ACF system was simultaneously spiked with the ES solution (Table S1). A magnetic stirrer agitated the water sample continuously to ensure a uniform distribution of analytes and the SS solution. To condition the filtration system, the tube connecting the points (a) and (b) in Figure 2 was rinsed, and the SPE-ACF system was conditioned with Milli-Q water.

The meltwater was eventually pumped through the SPE-ACF. After filtering the entire sample, the barrel and the aspiration system were washed with Milli-Q water. The wash water from both the barrel and the aspiration system was also collected in the SPE-ACF system.

After being used to filter the water, the adsorbent was extracted in Soxhlet with toluene. The extract was concentrated, and the clean-up was performed (see Section 3.2). Prior to GC-MS analysis, the sample was concentrated and spiked with the IS solution. For further details, refer to Section 3.2.

Each step of the procedure was evaluated using a specific isotopically labeled standard: the SS solution was used during the sampling, the ES solution in the extraction step, and the IS solution before injection. By assessing the recoveries across all analytical steps, it is possible to determine the extent of compound losses at each step and guarantee the method selectivity. The use of isotopically labeled solutions for each step is crucially important in these kinds of studies, as the analytes of interest are usually present in the samples at trace level concentrations. For this reason, the EPA methods include the control of a sample at various steps: (i) the percentage recovery (%R) of the extraction solution is evaluated (ES vs. IS) to ensure it falls within required ranges for the sample to be considered “acceptable”; (ii) the %R of the sampling solution (SS vs. ES; Equation (1)) is assessed to ensure it meets the method acceptability requirements for the sample to also be considered “quantifiable”. Therefore, it is necessary to evaluate, for each individual sample, if %Rs fall within the ranges of the methods.

The %Rs were compared to the ranges defined by EPA 1668B and 1699 methods [32,37] (Tables S5 and S6).

### 2.3. Validation of the Methodology Using EPA 1699 Pesticides and EPA 1668B PCBs in Water

According to EPA Method 1699, any modification to the method requires the laboratory to perform Initial Precision and Recovery (IPR) testing. If the modification affects the method’s detection limit, the lab must demonstrate that the new Method Detection Limits (MDLs) are either less than one-third of the regulatory compliance level or less than the MDLs specified in the method, whichever is greater. Additionally, EPA 1699 mandates performing literature reviews to support and justify any changes made to the method, and it requires that records of all method modifications be kept.

This includes the results from all quality control (QC) tests conducted to compare the modified method with the original, covering the following areas: (a) Calibration; (b) Calibration verification; (c) Initial precision and recovery; (d) Labeled compound recovery; (e) Analysis of blanks; and (f) Accuracy [37].

The results defined by points (a) and (b), which refer to common good laboratory practices, were performed via programmed instrumental tunes and calibration kits consisting of a 5-point concentration curve injected 3 times. The results of points (c), (d), (e), and (f), which represent the true method validation, are described below, along with the procedures used to obtain them. Figure 1 shows a schematic representation of the points included in the QC test. The validation of the new extraction method serves as the second objective of this work, which will subsequently be integrated into the workflow for processing snow samples.

In this case, considering that the matrices came from atmospheric wet depositions, snow, and rainwater, the “IPR” section of EPA 1699 related to samples with low content of suspended solids concentration was chosen.

As recommended in EPA Method 1699 [37], IPR tests were carried out to ensure the method’s reliability and compliance with QA/QC standards; labeled compound recoveries, method blanks, and accuracy were included to assess method performance.

The IPR assessment for low water suspension content considers aliquots of 1 L of water without native components to which the Standard solution of native and labeled compounds is added in 1 mL of acetone, following the concentrations reported in Table S7 [37].

The samples containing the Standard solutions (both native and labeled), once extracted and subsequently eluted, should be concentrated to achieve the concentrations shown in Table S7. The labeled standard solution also contained the compound D_6_-α-HCH, which is not covered by EPA Method 1699. Consequently, there are no reference values for R%, nor specified quantities or acceptance intervals. To use D_6_-α-HCH as a reference, the values for ^13^C_6_-γ-HCH presented in Tables S6 and S7 were associated with it.

In this context, accuracy was determined as the average recovery rate of each labeled compound from the standard solutions (%R_SS_) added to the samples, along with the calculation of the relative standard deviation (RSD). The sampling efficiency represents the accuracy during the sampling step and was assessed by the recovery rate of the SS solution (%R_SS_) added before sampling (Equation (1)).

While for the IPR tests, Equation (2) was also applied in which the R% of natives (natives vs. ES) were evaluated.

Processing four samples under identical conditions allowed us to assess the method’s accuracy, precision and repeatability (using the RSD). Accuracy was assessed by recovering the added labeled standards. As for the laboratory blank, it allowed us to check potential contamination from reagents, glassware, and laboratory instruments. The use of labeled standards in every step of the method (including from reagents, glassware, or laboratory equipment) ensured the monitoring of efficiency. 

### 2.4. SPE-ACF Extraction—Validation Method Based on IPR Tests

The SPE-ACF setup arises from the concept that the water–particulate system cannot be separated for a complete analysis. Consequently, the combined collection membranes (QFF and ACF) cannot be analyzed separately because the water–particulate system is in a continuous state of re-equilibrium. Therefore, the use of a QFF/ACF/QFF sandwich disc was the most analytically appropriate approach for quantitative analysis, as long as the breakthrough volume did not exceed that. Since it was hard to predict the volume of meltwater obtained during snow sampling (due to its dependence on snow consistency), a large volume of water was used to test the breakthrough under extreme conditions. For this purpose, the tests were carried out on 15 L of water, which is, according to the literature, the average volume obtained from sampling compacted snow in remote areas using 60–40 L barrels.

First, four IPR laboratory recovery tests were performed, as described in the above section. Table 1 shows the average recovery rates ($\bar{R}\%$) and the relative standard deviation (RSD, *n* = 4), as well as the corresponding accepted range and RSD from the EPA 1699 method. 

The data obtained met the EPA 1699 acceptance criteria, confirming the satisfactory performance of the method and validating the use of SPE-ACF in water volumes up to 15 L (Tables S5 and S6). In addition, the high recovery rates and the low corresponding RSD of labeled HCH isomers (Table 1) indicate that the analytes were completely collected from the sampling vessel without losses due to surface adhesion on the walls.

The RSD values for the pesticides and PCBs presented in Table 1 indicate a high level of precision and reproducibility for the SPE-ACF method. Specifically, the RSD percentages fall between 1% and 8%, which are significantly lower than the acceptable limit of 30% set by the EPA Method 1699. This low RSD suggests that the method consistently produces reliable results across multiple tests, even working with large sample volumes of up to 15 L. Furthermore, the results for the labeled HCH isomers demonstrate that the analytes were effectively collected from the sampling vessel with minimal losses due to surface adhesion. Overall, these findings underscore the robustness of the SPE-ACF method in environmental applications, indicating that it can maintain high analytical performance even under challenging conditions.

The IPR described by the EPA 1699 method did not require the use of an ES solution, but only a mix of native and SS solutions added to the water. Nonetheless, given the large volumes of water used, the ES solution was added to the validation procedure to evaluate the extraction step. In particular, it was evaluated whether the SPE-ACF system could adsorb pesticides without loss or if the volumes used exceeded the breakthrough volume. For this purpose, as mentioned above, an ES solution (Table S1) was added onto the SPE-ACF system before filtering the water with SPE-ACF at time 0 (t = 0). Table 2 shows the average recovery rates ($\bar{R}\%$) and the relative standard deviation (RSD) for the four IPR tests, along with the corresponding limits from the EPA 1668B method.

The use of multiple isotopically labeled standards is crucial for assessing recovery rates, losses, and contamination that occur during the analytical steps of sampling and extraction proposed in this study. Their use is necessary to address the requirements of EPA Method 1699 by justifying any modifications through the recovery percentages (R%) and relative standard deviations (RSD). In this context, given the absence of control in any previous work, the employment of these standards ensures the repeatability (low RSD) and accuracy (high R%) of the analytical procedures.

The IPR RSD observed in this study is better than that reported in the EPA Method 1668B (Table S5). This discrepancy is likely attributable to the advancements in instrumentation technology used during the experimental tests. Specifically, the EPA values from 2008 were probably obtained using low-resolution instruments, whereas this study utilized a high-resolution instrument (GC-Orbitrap). The use of a high-resolution instrument enables a greater number of acquisitions during analysis, resulting in more precise peak area definition and reduced uncertainty in quantification. This consideration represents further confirmation of the goodness of the proposed analytical method.

### 2.5. Application of the Methodology to Real Samples

**Figure 3:**
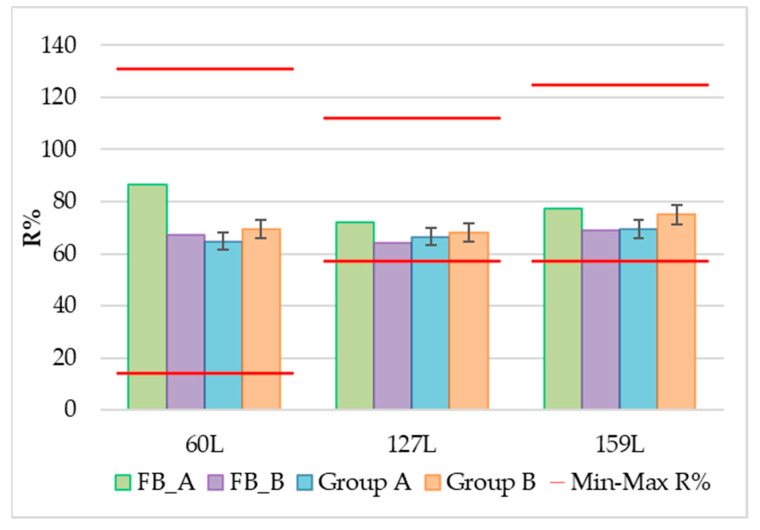
Average recovery rates (%R) of ^13^C-PCBs ES solution for four samples and their respective blanks from each group. Red lines indicate maximum and minimum %R according to EPA 1668B method.

**Figure 4:**
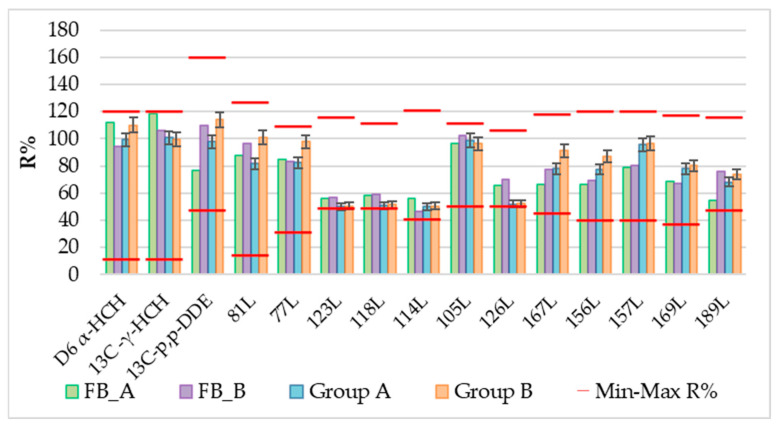
Average recovery rates (%R) of ^13^C-PCBs and pesticides SS solution for four samples and their respective blanks from each group. Red lines indicate maximum and minimum %R according to EPA 1699 and 1668B methods.

The validated extraction method for PCBs and pesticides was then applied in experimental campaigns to evaluate the linearity of the method considering two different scenarios: high and low concentrations. To overcome the analytical detection limit, the two groups of samples were collected in a remote area and in a urban area. Additionally, the matrix effect was assessed, considering the presence of other compounds in the snow. Two groups of samples were collected, and the meltwater volumes obtained were determined: an average of 17.1 ± 1.5 L for group A samples (remote area, 50 L barrel) and an average of 0.6 ± 0.1 L for group B samples (urban site, 1 L bottle). Each sample was treated according to the optimized analytical method. The recovery rates for the ES solution added to the ACF-SPE prior to the meltwater extraction and for the SS solution spiked in the samples in comparison are shown in Figure 3 and Figure 4. The %Rs ranges as required by the EPA methods are reported in Tables S8 and S9. The recovery rate was evaluated considering the average of the four samples for each group.

Both the sampling and the clean-up recovery rates meet the requirements of the EPA 1668B and 1699 methods. Due to the low concentration of the analytes and the potential for interfering compounds, the matrix effect evaluation in real Arctic samples has a key role. We consider the general control criteria of the tested ACF method to be acceptable since the %Rs of the two groups of samples and blanks were within the given limits. The use of three labeled solutions (SS, ES, and IS) during analyte extraction from Milli-Q water and melted snow, before SPE-ACF water filtration, and before injection allowed for sample-specific method validation.

Since the aim of this work was not to evaluate the environmental impact of the native pollutants under study, the quantitative data (pg/L) of the chlorinated pesticides for the two groups A and B will be presented as averages of the four samples (Table 3). In the context of method validation, the standard deviation of the data across the four samples is more significant than the quantitative value of the analyte itself. Nonetheless, a brief explanation of the results will be provided. Since the results were all below the detection limit, PCBs are not listed in the table.

The average concentrations detected in the two sample groups were different, as shown in Table 3. It confirmed that the sampling location is critical in determining the amount of snow to be collected. Group B samples were collected in the urban area of Longyearbyen, and 1 L of snow was sufficient to determine all investigated pesticides; in the remote area (group A samples), 50 L of snow was required.

The concentrations of α-HCH and γ-HCH in group A are 10 times lower than those reported in the literature [39]; it is reasonable to assume that the environmental conditions under which the snow was collected influenced these results. The compounds under investigation are photosensitive, and since there was 24 h of daylight in that region during the sampling period (May), compound degradation is assumed. Since the sampling period is the same, the degradation effects of the compounds were also assumed for Group B samples as well, suggesting that there could be a local source and the measured value could probably be underestimated.

The observed differences in average concentrations of organochlorine pesticides between Group A (remote area) and Group B (urban area) samples are noteworthy. Specifically, the significantly higher concentrations in the urban site highlight the potential influence of local pollution sources, which may be contributing to elevated pesticide levels. In contrast, the lower concentrations in the remote area suggest that long-range transport of these compounds, rather than local sources, is a primary factor affecting their presence. This finding aligns with previous studies that have demonstrated the persistence of organochlorine pesticides in the Arctic environments, where they can accumulate over time due to their resistance to degradation. The results also indicate that while the extraction method is effective in capturing these analytes, the environmental context in which the samples are collected plays a crucial role in determining the concentration observed. Further investigations are warranted to assess the long-term trends and potential ecological impacts of these pollutants in both remote and urban Arctic ecosystems.

To further delve into the aspect of the sources, the α/γ-HCH ratio was evaluated. The α/γ-HCH ratio can vary depending on the sources and the environmental context. Natural HCH contamination, such as biogenic production or geological processes, typically results in an α/γ-HCH ratio of close to 1. If the contamination is primarily from anthropogenic sources, the α/γ-HCH ratio can deviate from 1 [40]. A higher α/γ ratio may indicate recent use or release of technical-grade HCH, especially lindane, which is rich in γ isomer. The γ-HCH has been used in the past both as “technical HCH”, containing a mixture of isomers, and pure γ-HCH, known as lindane [41,42]. The α/γ-HCH ratio in technical HCH is about 4–5, while it is much less than 1 in lindane [43]. The average ratio is 1.8 and 0.6 for A and B groups, respectively. Given what has been said regarding the photodegradation of α/γ-HCH, definitive conclusions are impossible to make, although it appears evident that the sources were different.

The evaluation of the α/γ-HCH ratio provides critical insights into the sources and historical usage patterns of HCH isomers in the studied areas. A higher ratio in Group A indicates potential anthropogenic influences, possibly reflecting past agricultural practices or local industrial activities. Conversely, the lower ratio in Group B could suggest more recent or localized use, consistent with urban pollution patterns. The findings emphasize the need for comprehensive monitoring programs that can assess not only the current presence of these contaminants but also their sources and the potential risks they pose to both human health and wildlife. As climate change continues to alter environmental conditions in the Arctic, understanding the dynamics of these pollutants will be essential for effective management strategies and policy formulation aimed at mitigating their impacts.

As highlighted in several papers [44,45], HCB, along with pp-DDE and o,p-DDT, tends to accumulate in non-source regions such as the Arctic, given their persistence [13]. The difference in concentrations between Group A and B samples can be attributed to the sources: long-range transport in Site A and local pollution in Site B. Given the high concentrations, particularly of HCB, it cannot be ruled out that the anthropic activities are a source and that Group A samples were unaffected by them [46].

## 3. Material and Methods

### 3.1. Chemicals and Reagents

Three isotopically labeled Standard solutions, provided by Wellington Laboratories (Guelph, ON, Canada), were used: the Sampling Standard solution (SS solution), a mix of ^13^C-PCBs (WP-LCS) and labeled pesticides (D_6_-α-HCH, ^13^C_6_-γ-HCH, ^13^C_12_-p,p-DDE, ^13^C_6_-HCB); the Extraction Standard solution (ES solution) containing only ^13^C-PCB (P48-SS) to which the pesticides are referred to; and the Injection Standard solution (IS solution) added before instrumental analysis (^13^C-PCB WP-ISS). A solution containing a mixture of native pesticides (o,p′-DDT, p,p′-DDE, α-HCH, γ-HCH, and Hexaclorobenzene (HCB)) was used in addition to the SS solution to validate the method (Initial Precision Recovery Test, IPR). Comprehensive information about the Standard solutions can be found in the Supplementary Materials (Table S1).

PCBs and pesticide calibration standard curve with 5 concentration points (0.1–800 pg/µL) were provided by Wellington Laboratories (Guelph, ON, Canada).

Acetone, methanol, dichloromethane (DCM), toluene, and n-hexane (HEX) Super Purity Solvents grade were supplied by Romil (Cambridge, UK).

### 3.2. Extraction and Analysis

The SPE-ACF system consisted of a sandwich structure composed of a QFF/ACF/QFF with a diameter of 102 mm, using quartz filters (Whatman, Maidstone, Kent, UK)) and an ACF filter provided by Chemical Research 2000 srl, Rome, Italy. A 1000 pg amount of the SS solution (Table S1) mixed with 1 mL of acetone was spiked into the melted snow sample, and the SPE-ACF system was simultaneously spiked with 1000 pg of the ES solution (Table S1). To condition the extraction setup, the SPE-ACF system was rinsed with 500 mL of Milli-Q water (MilliporeSigma, Burlington, MA, USA). The flow rate used for filtering the snow was 1 L/min. Following sample filtration, the barrel was washed twice with 250 mL of Milli-Q water, and an additional 100 mL rinse was applied to the aspiration system, with all wash solutions collected within the SPE-ACF system. After filtration, the adsorbent was extracted in a 100 mL Soxhlet apparatus with TOL for 36 hours. The resulting extract was then concentrated via rotary evaporation and reduced to 0.5 mL under gentle N₂ flow. For sample cleanup, the extract was applied to a basic alumina column and eluted with 15 mL of HEX:DCM 94:6 (*v*/*v*) solution. Before analysis on the GC-Orbitrap (Thermo Fisher Scientific, Waltham, MA, USA), the sample was concentrated to approximately 200 µL and spiked with 1000 pg of the IS solution. Detailed information on the GC column, GC–MS settings, SIM, and full-scan methods is provided in Tables S3 and S4 of the Supplementary Materials.

This procedure incorporated isotopically labeled standards at each stage—SS during sampling, ES during extraction, and IS before injection—to monitor recoveries and ensure analyte retention, essential for accurate trace-level analysis. The recovery percentage for each step (%R) was calculated to evaluate potential losses and ensure method selectivity, with %R_SS_ for the SS solution and %R_ES_ for the ES solution.

(1) $$\%R_{SS}=\frac{RRF}{100}\times \frac{A_{SS}}{A_{ES}}\times \frac{Q_{ES}}{Q_{SS}}$$

The %R_SS_ is the recovery rate of the SS solution added before sampling. RRF is the relative response factor that quantifies the mass spectrometer response to a known amount of an analyte relative to a known amount of a ^13^C-labeled internal standard and is calculated using the calibration curve. A_SS_ and A_ES_ represent the areas of signal relative to the m/z ratio used for the quantification of SS and ES labeled standards, respectively, while Q_SS_ and Q_ES_ represent the amount injected. To calculate %R_ES_, parameters in Equation (1) that refer to the SS are replaced with those of the ES, while those of the ES are substituted with those of the IS.

### 3.3. IPR Test

To meet EPA requirements for Initial Precision and Recovery (IPR), recovery tests and laboratory blanks are performed as follows:

The laboratory recovery test involved four pre-cleaned barrels (cleaned with methanol, acetone, and DCM), each filled with 15 L of Milli-Q water. These barrels were spiked with native pesticides (α-HCH, γ-HCH, p,p′-DDE, and o,p′-DDT) and the SS solution (see Table S1), combined with 1 mL of acetone directly into the Milli-Q water.

The laboratory blank was prepared by filling a separate pre-cleaned (methanol, acetone, and DCM) barrel with 15 L of Milli-Q water, which was then spiked only with the SS solution (Table S1).

Both the laboratory recovery tests and the laboratory blank were processed using the full analytical method, as detailed in Section 3.2.

For native compounds, in the Equation (1), A_SS_ is replaced with the area of the native compound and Q_SS_ with their concentrations (Equation (2)).

(2) $$\%R_{n}=\frac{RRF}{100}\times \frac{A_{n}}{A_{ES}}\times \frac{Q_{ES}}{Q_{n}}$$

Since the validation of ACF as an adsorbent for the analysis of PCBs in water has already been carried out [35], further investigation was carried out to validate it for pesticides. Since the two methods do not conflict, PCB validation was also repeated. For this reason, the QC criteria of %R for IPR and RSD for PCBs are also reported, and the SS solution used contained labeled congeners of both pesticides and PCBs (WP-LCS).

### 3.4. Field Experiment—Sample Sites

**Figure 5:**
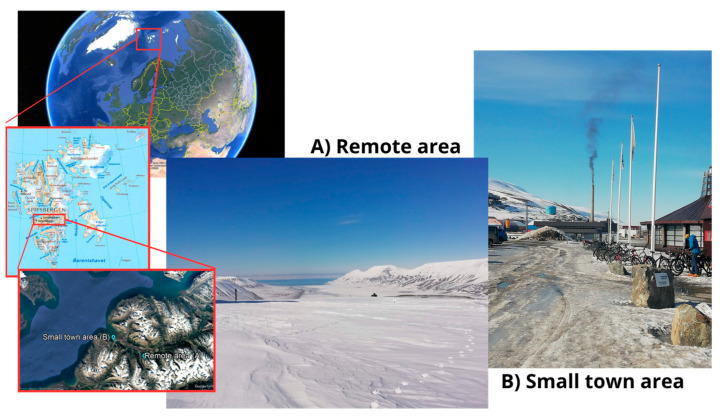
Sampling sites: Spitsbergen island in Svalbard. (**A**) Remote area, Group A samples; (**B**) Small town area, Group B samples.

Two sets of four snow samples each were collected from Spitsbergen, an island of the Svalbard archipelago in the European Arctic (Figure 5). Group A samples were collected in a remote area (536 m above sea level) in 50 L barrels. Group B samples were collected in the urban area of Longyearbyen (16 m above sea level) in 1 L glass bottles, as higher concentrations were expected, given the urban site. One field blank (FB), consisting of 1 L of MilliQ water, was also collected for each group (FB_A and FB_B) to assess potential contaminations. FBs were brought into the field and exposed to the atmosphere during the first sample collection of each group, following the same procedure as the snow samples. This aligns with the EPA’s requirement for field blanks, which should be composed of a matrix as similar as possible to the actual sample and follow the same procedure. Citing the official reference: “*The matrix for the method blank must resemble the sample matrix for the batch, such as a 1-L reagent water blank. Spike 1.0 mL of the labeled spiking solution into the method blank. Prepare, extract, clean up, and concentrate the method blank*”.

To prevent operator contamination, samples were collected as described in Section 2.1 [32].

**Figure 6:**
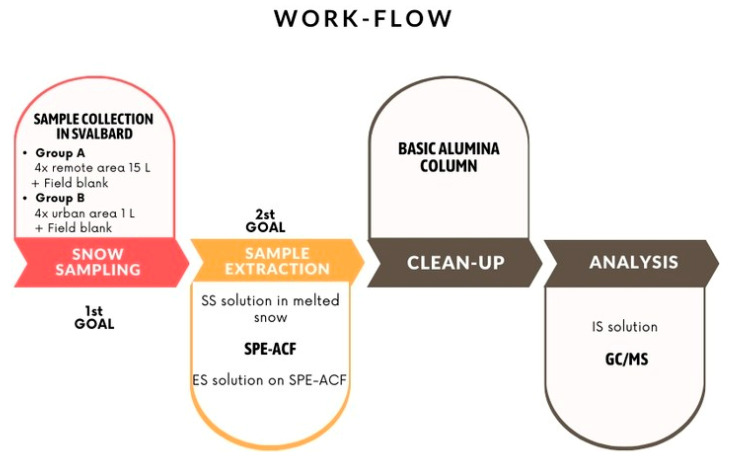
Workflow for snow analysis. Both objectives are incorporated into the procedure for analyzing pesticides and PCBs in snow. The method was applied to real samples collected in Svalbard.

The sample groups were analyzed following the workflow for snow sample analysis presented in Figure 6.

## 4. Conclusions

There are currently no guideline methods for the sampling and laboratory treatment of snow samples. There are no procedures in agreement among the different research groups. It has been critically highlighted how the sampling step (very often neglected) is fundamental, both for data collection and for potential contamination due to operators. The extraction method proposed here, which uses an ACF-based SPE for organochlorine pesticides and PCBs, has been validated in the laboratory through an IPR test (according to EPA 1699 and 1668B methods for pesticides and PCBs, respectively). Subsequently, the outlined method (from sampling to extraction, including the final analyses) was tested on the samples of Arctic surface snow, which were collected from Spitsbergen (Svalbard, Norwegian Arctic) in two distinct zones (remote and urban) in different volumes (1 L and 50 L of snow), and its efficiency was evaluated by the matrix effect. Among the various advantages of this method, the main one is the lower impedance of the sorbent material since the ACF used has zero impedance. It is certainly necessary to perform a more in-depth analysis to define specific environmental conclusions, but it can be argued that the proposed method is valid for both more concentrated snow samples (group B, low volumes) and samples containing compounds in trace (group A, high volumes). In conclusion, this work aims to become a starting point for the definition of specific guidelines and certified methods for sampling and analyzing snow.

## Figures and Tables

**Table 1:** Average recovery rates ($\bar{R}\%$) and relative standard deviation (RSD) of 4 IPR laboratory tests for pesticides and PCBs. (“L” means ^13^C_12_-labeled).

	Laboratory Test Results		EPA 1699 Accepted Range	
	$\mathrm{IPR}\bar{R}\%$	IPR RSD	$\mathrm{IPR}\bar{R}\%$	IPR RSD
α-HCH	87	8	55–108	30
γ-HCH	72	4	55–108	30
HCB	103	3	55–108	30
p,p′,-DDE	81	1	55–108	30
o,p′-DDT	90	8	55–108	30
D_6_-α-HCH	94	4	6–112	62
^13^C_6_-γ-HCH	98	5	6–112	62
^13^C_12_-p,p′-DDE	75	17	29–152	43
^13^C_6_-HCB	87	4	6–108	70
81L	78	15	57–100	33
77L	74	12	57–100	35
123L	84	6	66–103	32
118L	96	4	65–102	33
114L	86	5	57–100	41
105L	90	4	66–101	31
126L	83	5	67–100	29
167L	86	2	74–103	24
156L	92	2	61–100	35
157L	93	5	61–100	35
169L	95	4	66–103	33
189L	99	3	68–100	28

**Table 2:** Average recovery rates ($\bar{R}\%$) and relative standard deviations (RSD) of four IPR laboratory tests for PCBs, along with the acceptance criteria for R% and RSD% according to EPA Method 1668B.

	Laboratory Test Results		EPA 1668B Accepted Range	
	IPR $\bar{R}\%$	IPR RSD	IPR $\bar{R}\%$	IPR RSD
60L	77	19	43–106	63
127L	84	10	75–102	23
159L	88	11	78–117	30

**Table 3:** Average concentrations (pg/L) of organochlorine pesticides in the snow samples.

	Group A Remote Area	Group B Urban Area
	pg/L	pg/L
α-HCH	6.7 ± 0.6	68 ± 5
HCB	19.8 ± 0.7	1060 ± 30
γ-HCH	3.7 ± 0.2	105 ± 5
p,p′-DDE	115 ± 2	417 ± 5
o,p′-DDT	16 ± 1	355 ± 30

## Data Availability

The data that support the findings of this study are available from the corresponding author upon reasonable request.

## Supplementary Materials

The following supporting information can be downloaded at https://www.mdpi.com/article/10.3390/molecules29215111/s1, Table S1. Composition of the ^13^C standards for PCBs, ^13^C and D for pesticides, and native pesticides; Table S2. Sampling details for Group A (a) and Group B (b); Table S3. GC-Orbitrap conditions; Table S4. Inclusion List of PCBs and Pesticides target SIM; Table S5. QC Acceptance criteria for IPR, RSD and labelled compounds in samples. Table Source: EPA Method 1668B (2008); Table S6. QC acceptance criteria for IPR and samples based on a 20 µL extract final volume. Table Source: EPA Method 1699 (2007); Table S7. Concentrations of native and labelled pesticides in spiking solutions and final extracts. Table Source: EPA Method 1699 (2007); Table S8. Maximum and minimum %R according to EPA 1668B method for 13C-PCBs ES Solution; Table S9. Maximum and minimum %R according to EPA 1699 and 1668B method for 13C-PCBs and pesticides SS Solution.
