# Supplementary material for: Mastering Snow Analysis: Enhancing Sampling Techniques and Introducing ACF Extraction Method with Applications in Svalbard
Source: Molecules. 2024 Oct 29;29(21):5111. doi: 10.3390/molecules29215111 (PMC11547376; doi:10.3390/molecules29215111)
Supplement: Supplementary file 1 [file molecules-29-05111-s001.zip › molecules-3237084-supplementary.pdf]

Supplementary Materials

Table S1. Composition of the ^{13}C-labeled standards for PCBs, ^{13}C- and D-labeled for pesticides, and native pesticides.

Name	Acronym	P-48SS (pg/µL)	WP-LCS (pg/µL)	WP-ISS (pg/µL)	Labeled pesticides (pg/µL)	Native pesticides (pg/µL)
2,3,4,4′-Tetra[$^{13}C_{12}$]B	60L	100	-	-	-	-
3,3′,4,5,5′-Penta[$^{13}C_{12}$]B	127L	100	-	-	-	-
2,3,3′,4,5,5′-Hexa[$^{13}C_{12}$]B	159L	100	-	-	-	-
3,4,4′,5-Tetra[$^{13}C_{12}$]B	81L	-	1000	-	-	-
3,3′,4,4′-Tetra[$^{13}C_{12}$]B	77L	-	1000	-	-	-
2′,3,4,4′,5-Penta[$^{13}C_{12}$]B	123L	-	1000	-	-	-
2,3′,4,4′,5-Penta[$^{13}C_{12}$]B	118L	-	1000	-	-	-
2,3,4,4′,5-Penta[$^{13}C_{12}$]B	114L	-	1000	-	-	-
2,3,3′,4,4′-Penta[$^{13}C_{12}$]B	105L	-	1000	-	-	-
3,3′,4,4′,5-Penta[$^{13}C_{12}$]B	126L	-	1000	-	-	-
2,3′,4,4′,5,5′-Hexa[$^{13}C_{12}$]B	167L	-	1000	-	-	-
2,3,3′,4,4′,5-Hexa[$^{13}C_{12}$]B	156L	-	1000	-	-	-
2,3,3′,4,4′,5′-Hexa[$^{13}C_{12}$]B	157L	-	1000	-	-	-
3,3′,4,4′,5,5′-Hexa[$^{13}C_{12}$]B	169L	-	1000	-	-	-
2,3,3′,4,4′,5,5′-Hepta[$^{13}C_{12}$]B	189L	-	1000	-	-	-
2,3′,4′,5-Tetrachloro[$^{13}C_{12}$]B	70L	-	-	1000	-	-
2,3,3′,5,5′-Penthachloro[$^{13}C_{12}$]B	111L	-	-	1000	-	-
2,2′,3,4,4′,5′-Hexachloro[$^{13}C_{12}$]B	138L	-	-	1000	-	-
2,2′,3,3′,4,4′,5-Heptachloro[$^{13}C_{12}$]B	170L	-	-	1000	-	-
α-Hexachlorocyclohexane-D_6	D_6-α-HCH				2600	
γ –Hexachlorocyclohexane-$^{13}C_6$	$^{13}C_6$-γ-HCH				2600	
$^{13}C_{12}$-p,p′- Dichlorodiphenyldichloroethylene	$^{13}C_{12}$-p,p-DDE				1600	
$^{13}C_6$-Hexaclorobenzene	$^{13}C_6$-HCB				1800	
o,p′- Dichlorodiphenyldichloroethylene	o,p′-DDT					600
p,p′- Dichlorodiphenyldichloroethylene	p,p′-DDE					600
α-Hexachlorocyclohexane	α-HCH					1200
γ –Hexachlorocyclohexane, Lindane	γ-HCH					1200
Hexaclorobenzene	HCB					800

Table S2. Sampling details for Group A (a) and Group B (b).

a.

**Parameters**	**Samples Group A**			
	**1**	**2**	**3**	**4**
Volume	50 L	50 L	50 L	50 L
GPS location	78°08′49.8″N 16°01′57.0″E	78°08'49.7"N 16°01'52.1"E	78°08'50.2"N 16°01'54.5"E	78°08'49.6"N 16°01'56.0"E
Sampling area	536 m a.s.l. 100x130 cm area 5±1 cm depth	536 m a.s.l. 108x120 cm area 5±1 cm depth	536 m a.s.l. 90x145 cm area 5±1 cm depth	536 m a.s.l. 105x124 cm area 5±1 cm depth
Sampling data	May 10th 2019, 12:13 pm	May 10th 2019, 12:59 pm	May 10th 2019, 1:38 pm	May 10th 2019, 2:15 pm
Surface temperature	-3.0±0.1 °C	-3.2±0.1 °C	-2.9±0.1 °C	-3.0±0.1 °C
In-depth temperature	-7.0±0.1 °C	-7.1±0.1 °C	-6.8±0.1 °C	-7.1±0.1 °C

b.

**Parameters**	**Samples Group B**			
	**1**	**2**	**3**	**4**
Volume	1 L	1 L	1 L	1 L
GPS location	78°13′01.3″N 15°36′41.8″E	78°13'01.3"N 15°36'41.8"E	78°13'00.4"N 15°36'40.1"E	78°13'02.0"N 15°36'41.2"E
Sampling area	16 m a.s.l. 44x12 cm area 5±1 cm depth	16 m a.s.l. 41x13 cm area 5±1 cm depth	16 m a.s.l. 51x10cm area 5±1 cm depth	16 m a.s.l. 36x15 cm area 5±1 cm depth
Sampling data	May 10th 2019, 4:50 pm	May 10th 2019, 5:12 pm	May 10th 2019, 5:35 pm	May 10th 2019, 5:52 pm
Surface temperature	2.3±0.1 °C	2.5±0.1 °C	2.0±0.1 °C	2.2±0.1 °C
In-depth temperature	-3.1±0.1 °C	-2.9±0.1 °C	-3.3±0.1 °C	-3.0±0.1 °C

Table S3. GC-Orbitrap conditions.

GC- Orbitrap MS ThermoScientific Trace 1310, Exactive GC. Column Agilent DB-XLB, Length: 60 m; I.D. 0.25mm; Film: 0.25 µm. Carrier gas He.

PTV mode	CT Splitless w/Surge
Injection Initial T °C	200
Hold (min)	-
Transfer initial ramp (°C/min)	-
Final T (°C)	-
Isotherm (min)	-
Cleaning ramp (°C/min)	-
Final T (°C)	-
Isotherm (min)	-
Splitless time (min)	0.80
Splitless flow (mL/min)	100
Carrier mode	Programmed Flow
Flow (mL/min)	1.200
Hold (min)	30.00
Rate (mL/min)	0.400
Flow (mL/min)	1.300
Hold (min)	30.00

Initial temperature (°C)	130
and hold (min)	1
Initial ramp (°C/min)	30.00
Final T (°C)	170
Isotherm (min)	5.00
Second hold (°C/min)	7.00
Final T (°C)	190
Isotherm (min)	2.00
Third hold (°C/min)	5.00
Final T (°C)	245
Isotherm (min)	12.00
Final hold (°C/min)	7.00
Final T (°C)	325
Isotherm (min)	10.00

Source temperature °C	300
Transfer line °C	280
Transfer line 1 and 2 °C	280

Electron energy (eV)	70
Emission current (μA)	50
Ionization mode	EI+

Resolution	60,000
AGC Target	1e6
Maximum IT	Auto
Scan Range	50 to 500 m/z

Resolution	30.000
AGC Target	5e5
Maximum IT	auto
Isolation window m/z	10.0

Table S4. Inclusion List of PCBs and Pesticides target SIM. "" means ^{13}C- and D-labeled.*

**Inclusion List of Target SIM**				
**Mass [m/z]**	**Polarity**	**Start [min]**	**End [min]**	**Comment**
185	Positive	7.00	22.00	α-HCH* γ-HCH*, α-HCH, γ-HCH
235	Positive	22.00	39.00	o,p'-DDT
248	Positive	22.00	39.00	p,p'-DDE
253	Positive	10.00	20.00	PeCB* PeCB
258	Positive	22.00	39.00	o,p'-DDE*
266	Positive	15.00	26.00	TCN
287	Positive	10.00	20.00	HCB* HCH
292	Positive	24.00	36.00	TetraCB
304	Positive	22.00	36.00	TetraCB*
326	Positive	25.00	40.00	PentaCB
338	Positive	23.00	40.00	PentaCB*
360	Positive	30.00	47.00	HexaCB
372	Positive	30.00	47.00	HexaCB*
394	Positive	36.00	47.00	HeptaCB
406	Positive	36.00	47.00	HeptaCB*

Table S5. QC Acceptance criteria for IPR, RSD and labeled compounds in samples. Table Source: Method 1668B (2008) [32]

	**IPR Recovery (%)**	**RSD**	**Recovery of Labeled Compounds in Samples (%)**
		**SS Solution**	
81L	57 - 100	33	14-127
77L	57 - 100	35	31-109
123L	66 - 103	32	49-116
118L	65 - 102	33	49-111
114L	57 - 100	41	41-121
105L	66 - 101	31	50-111
126L	67 - 100	29	50-106
167L	74 - 103	24	45-118
156L	61 - 100	35	40-120
157L	61 - 100	35	40-120
169L	66 - 103	33	37-117
189L	68 - 100	28	47-116
		**ES Solution**	
60L	43-106	63	14-131
127L	75-102	23	57-112
159L	78-117	30	57-125

Table S6. QC acceptance criteria for IPR and samples based on a 20 μL extract final volume. Table Source: EPA Method 1699 (2007) [37]

**Pesticide**	**IPR $\bar{R}$% Limits**	**IPR RSD**	**Recovery in samples (%)**
o,p'-DDT	55 - 108	30	
p,p'-DDE	55 - 108	30	
α-HCH	55 - 108	30	
γ-HCH (Lindane)	55 - 108	30	
Hexaclorobenzene (HCB)	55 - 108	30	
**SS Solution**			
D_6-α-HCH	6 - 112	62	11 - 120
$^{13}C_6$-γ-HCH	6 - 112	62	11 - 120
$^{13}C_{12}$-p,p-DDE	29 - 152	43	47 - 160
$^{13}C_6$-HCB	6 - 108	70	5 – 120

Each step of the procedure was evaluated using a specific isotopically labeled standard: the SS solution was used during the sampling, the ES solution in the extraction step, and the IS solution before injection. By assessing the recoveries across all analytical steps, it is possible to determine the extent of compound losses at each step and guarantee the method selectivity.

Table S7. Concentrations of native and labeled pesticides in spiking solutions and final extracts. Table Source: EPA Method 1699 (2007) [37].

	**Spiking solution (pg/μL)**	**In 20 μL extract (ng/mL; pg/μL)**
Hexachlorobenzene (HCB)	800	40
α-HCH	1200	60
γ-HCH (Lindane)	1200	60
2,4'-DDT	600	30
4,4'-DDE	600	30
$^{13}C_6$-HCB	1800	90
D_6-α-HCH	*2600*	*130*
$^{13}C_6$-γ-HCH	2600	130
$^{13}C_{12}$-p,p-DDE	1600	80

Table S8. Minimum and maximum %R according to EPA 1668B method for ^{13}C-PCBs ES Solution.

	**Minimum %R**	**Maximum %R**
60L	14	131
127L	57	112
159L	57	125

Table S9. Minimum and maximum %R according to EPA 1699 and 1668B methods for ^{13}C- and D-labeled PCBs and pesticides SS Solution.

	**Minimum %R**	**Maximum %R**
D_6 α-HCH	11	120
^{13}C -γ-HCH	11	120
^{13}C-p,p-DDE	47	160
81L	14	127
77L	31	109
123L	49	116
118L	49	111
114L	41	121
105L	50	111
126L	50	106
167L	45	118
156L	40	120
157L	40	120
169L	37	117
189L	47	116
